# USP53 Affects the Proliferation and Apoptosis of Breast Cancer Cells by Regulating the Ubiquitination Level of ZMYND11

**DOI:** 10.1186/s12575-024-00251-4

**Published:** 2024-07-23

**Authors:** Xiangchao Meng, Hongye Chen, Zhihui Tan, Weitao Yan, Yinfeng Liu, Ji Lv, Meng Han

**Affiliations:** 1 Breast Disease Diagnosis and Treatment Center, First Hospital of Qinhuangdao, Qinhuangdao, 066000 P. R. China; 2 Department of General Surgery, First Medical Center, Chinese PLA General Hospital, Beijing, 100000 P. R. China

**Keywords:** USP53, ZMYND11, Breast cancer, Ubiquitination, Deubiquitination, Proliferation, Apoptosis

## Abstract

Breast cancer is the most common female malignancy worldwide. Ubiquitin-specific peptidase 53 (USP53) has been shown to exert cancer-suppressing functions in several solid tumors, but its role and the underlying mechanism in breast cancer has not been clearly elucidated. Therefore, we have carried out a series of detailed studies on this matter at the levels of bioinformatics, clinical tissue, cell function and animal model. We found that USP53 expression was downregulated in breast cancer specimens and was negatively correlated with the clinical stages. Gain- and loss-of-function experiments demonstrated USP53 inhibited proliferation, clonogenesis, cell cycle and xenograft growth, as well as induced apoptosis and mitochondrial damage of breast cancer cells. Co-immunoprecipitation data suggested that USP53 interacted with zinc finger MYND-type containing 11 (ZMYND11), and catalyzed its deubiquitination and stabilization. The 33–50 amino acid Cys-box domain was key for USP53 enzyme activity, but not essential for its binding with ZMYND11. The rescue experiments revealed that the anti-tumor role of USP53 in breast cancer cells was at least partially mediated by ZMYND11. Both USP53 and ZMYND11 were prognostic protective factors for breast cancer. USP53-ZMYND11 axis may be a good potential biomarker or therapeutic target for breast cancer, which can provide novel insights into the diagnosis, treatment and prognosis.

**Supplementary Information:**

The online version contains supplementary material available at 10.1186/s12575-024-00251-4.

## Keypoints

1. USP53 has been shown to play an anticancer role in a variety of solid malignancies, but its role in breast cancer and its mechanism remain unclear.

2. Our results suggested that USP53 was low expressed in breast cancer and negatively correlated with TNM stage; USP53 suppressed proliferation and triggered apoptosis of breast cancer cells; USP53 inhibited breast cancer in vitro and in vivo by deubiquitinating ZMYND11.

3. USP53-ZMYND11 axis may be a potential breast cancer marker and therapeutic target.

**Supplementary Information:**

The online version contains supplementary material available at 10.1186/s12575-024-00251-4.

## Introduction

In 2020, breast cancer became the most prevalent cancer globally, with approximately 2.3 million new cases, making up 11.7% of all cancer diagnoses, surpassing lung cancer [1]. Although the mortality of breast cancer has dropped approximately 40% due to the high-quality prevention, early detection and advanced treatments, 684,996 new deaths each year have been still alarming [2]. It is beneficial for breast cancer patients to develop novel diagnosis markers and therapeutic targets.

Ubiquitination is a way of protein post-translational modification to regulate the stability and activity of the target proteins by covalently attaching ubiquitin to them [3]. Deubiquitination is the reverse reaction of the ubiquitination process, which mainly relies on the deubiquitinase (DUB) to split the ubiquitin and inactive ubiquitin precursors from the target proteins in order to maintain the free ubiquitin pool and protein degradation rate [4, 5]. More than 100 DUBs have been discovered so far, of which Ubiquitin-specific peptidases (USPs) constitute the largest and most complex family, with a total of 58 known members involved broadly in cell cycle, apoptosis, signaling pathway, DNA damage repair and other biological processes [6–8]. They are closely related to the occurrence and development of metabolic diseases, immune diseases, neurodegenerative diseases and malignant tumors [9]. USP53, a member of this family, is widely expressed in tissues of various human systems, participating in physiological activities such as cell apoptosis, glycolysis, neurotransmission, fat metabolism, bone formation and bone homeostasis [10–13]. Studies have found that USP53 is underexpressed and exerts tumor-suppressive effects in lung cancer, renal clear cell cancer, liver cancer and esophageal cancer [14–17]. However, whether and how USP53 affects the malignant phenotype of breast cancer has not been clear. Bioinformatics analysis showed that USP53 expression was down-regulated in breast cancer tissues compared to normal breast tissues, and patients with low expression of USP53 had worse survival outcomes than those with high expression. These evidences suggested that the downregulation of USP53 may be involved in development of breast cancer.

USP53 has been demonstrated to catalyze the deubiquitination of some proteins and block their degradation, such as cytochrome C and FKBP51 [14, 16]. Bioinformatics analysis found that there were correlations between the expression of USP53 and the activity of transcriptional coregulators in breast cancer, especially a significant positive correlation between the expressions of USP53 and zinc finger MYND-type containing 11 (ZMYND11), moreover, breast cancer patients with low expression of ZMYND11 also showed a worse prognosis than those with high expression. ZMYND11, also known as BS69 or BRAM1, plays an important role in chromatin remodeling, and its coding locus in chromosomes is a region often missing in malignant tumors [18, 19]. It is able to restrain specifically the expression of oncogenes, which is critical to regulate transcriptional programs necessary for tumor cell growth [20]. As a transcriptional co-suppressor of oncogenes, ZMYND11 is down-regulated in a variety of malignant tumors and exerts broad-spectrum tumor-suppressive effects [19, 21–23]. A Study confirmed that overexpression of ZMYND11 significantly inhibited the proliferation of MDA-MB-231 breast cancer cells in vitro and the growth of transplanted tumors in nude mice [23]. The above evidences suggest a possible synergistic effect between USP53 and ZMYND11, which means that ZMYND11 may mediate the function of USP53 in breast cancer.

In this article, we mainly investigate: (1) whether and how USP53 affects the malignant behaviors of breast cancer cells; (2) whether and how USP53 regulates the expression of ZMYND11.

## Materials and Methods

### Bioinformatics Analysis

RNAseq data for pan-cancer and corresponding normal tissues from TCGA and GTEx were downloaded from UCSC XENA website (https://xenabrowser.net/datapages/). The GSE10810 and GSE42568 datasets were downloaded from the GEO database (https://www.ncbi.nlm.nih.gov/geo/). The 3D structure diagram of USP53 was generated online using SWISS-MODEL (https://swissmodel.expasy.org/). The GEPIA2 website (http://gepia2.cancer-pku.cn/) contains RNAseq data for 9736 tumors and 8587 normal tissues as well as provides the corresponding python package for visual analysis. The Kaplan-Meier Plotter (http://kmplot.com/) online tool can assess the expression of all genes and their correlation with survival in more than 30,000 samples from 21 cancers.

The Limma package in R software was used to analyze the differential expression of USP family members in breast cancer and normal breast tissue from the TCGA database. Differentially expressed genes in breast cancer from GSE10810, GSE42568, TCGA and GEPIA2 were screened respectively, with the condition as log_2_FC < -1 and *p*adj < 0.05, then take the intersection of the four to obtain the down-regulated gene set.

GEPIA2 and Kaplan-Meier Plotter were used to analyze the differences of USP53 expression in the corresponding pan-cancer database. Stats and Car package was used to analyze USP53 expression differences and paired expression differences between 33 cancers and normal tissues from TCGA and GTEx databases. The results of the above four analyses were summarized to obtain the intersection cancer species.

The correlations between USP53 and all genes in breast cancer was analyzed by Pearson method, and then the conditions of |Cor| > 0.5 and *p*adj < 0.05 were set to screen for USP53 correlated gene sets. The GO&KEGG enrichment analysis of the selected gene sets was performed using the ClusterProfiler package. The pairwise similarity of the enriched items was calculated by Jaccard similarity index, and then cluster analysis was performed by Hclust function.

The diagnostic value of USP53 in breast cancer was analyzed using the pROC package. The Kaplan-Meier Plotter online tool was used to analyze the survival differences betwween patients with high and low expression of USP53/ZMYND11.

The Ggplot2 and VennDiagram packages were used to visualize the data analysis results above.

### Collection of Clinical Tissue Samples

Thirty pairs of fresh breast cancer and para-carcinoma tissues, and seventy-three paraffin breast cancer specimens, from November 2022 to October 2023, were collected from the Breast Disease Diagnosis and Treatment Center of First Hospital of Qinhuangdao. The breast cancer was diagnosed with pathological examination, and the tissues were removed with surgery. The paired breast cancer and para-carcinoma tissues were used for PCR and western blot detections, and the paraffin specimens were applied for immunohistochemistry staining. The informed consent was obtained from every patient. Experimental procedures were conducted according to the Declaration of Helsinki, which was approved by the Ethics Committee of Qinhuangdao First Hospital (approval number: 2022k009).

### Construction of Plasmid

To investigate the effect of USP53, its coding sequence was cloned into pcDNA3.1 vector, and the silencing fragment targeting USP53 was inserted into pRNA-H1.1/Neo vector. To explore the function details of USP53, its mutant sequence with 33–50 amino acid residues deficiency was synthesized and inserted into pcDNA3.1 plasmid. To verify the interaction between USP53 and ZMYND11, the USP53 coding sequence was inserted into pcDNA3.1-flag vector, and ZMYND11 coding sequence was inserted into pcDNA3.1-HA vector. The knockdown fragment targeting ZMYND11 was also inserted into pRNA-H1.1/Neo vector to suppress its expression. The USP53 or ZMYND11 overexpression plasmids were purchased from YouBio (Changsha, Hunan, China), and the knockdown sequences were synthesized by General Biol (Chuzhou, Anhui, China). The silencing sequences targeting USP53 or ZYMND11 were shown below:

shNC: GTTCTCCGAACGTGTCACGTTCAAGAGAACGTGACACGTTCGGAGAATTTTT

shUSP53-1: GGGATATCAGTGGTGTTAAATTCAAGAGATTTAACACCACTGATATCCTTTTT

shUSP53-2: GGGAAAGATGTTGTCTCCAATTCAAGAGATTGGAGACAACATCTTTCCTTTTT

shZMYND11: GGGCTATAGATCTTAATAAATTCAAGAGATTTATTAAGATCTATAGCCTTTTT

### Cell Culture and Treatment

Human immortalized mammary epithelial cell lines (MCF-12 A) and breast cancer cell lines (MCF-7, T47D, BT474, SKBR3, MDA-MB-453, MDA-MB-231) were purchased from iCell (Shanghai, China). MCF-12 A was cultured with the special medium provided by the manufacturer. MCF-7, T47D, BT474 and SKBR3 were cultured with MEM (SolarBio, Beijing, China). MDA-MB-453 and MDA-MB-231 were cultured with L-15 medium (Servicebio, Wuhan, Hubei, China). 10% FBS and 1% penicillin + streptomycin were added to MEM and L-15. The cells were grown at 37 C in a humidified atmosphere of 5% CO2.

Transfection was performed using LipofectamineTM 3000 reagent (Invitrogen, Carlsbad, CA, USA) in serum-free medium. The cells with transfection of USP53-overexpressed or -silenced vector were treated with G418 (500 µg/ml for MCF-7 cells and 600 µg/ml for MDA-MB-231 cells; Biosharp Life Science, Hefei, Anhui, China) at 24 h post transfection for 1–2 weeks, and the single cells were selected for continuous culture with G418 for 2 weeks. The surviving cells were considered as USP53-stably-overexpressed or silenced cells.

To intercept the protein synthesis, a translational inhibitor cycloheximide (CHX) (20 µg/ml; Aladdin, Shanghai, China) was applied, and a proteasome inhibitor MG132 (10 µM; Macklin Inc, Shanghai, China) was used to block protein degradation.

### Real-Time PCR

The concentration of total RNA was measured with the NANO 2000 ultraviolet spectrophotometer (Thermo Scientific, Wilmington, DE, USA) after being extracted from tissues or cells using the TRIpure Total RNA Extracting Kit (BioTeke, Beijing, China). BeyoRT-II M-MLV reverse transcriptase (Beyotime Institute of Biotechnology, Shanghai, China), with random primer as RT primer, was used to reverse transcribe RNA into cDNA (1 µg RNA from cells or 5 µg RNA from clinical specimens for one repeat). The reagent and instruments used in RT were RNase-free. Subsequently, the real-time quantificational PCR was performed to measure the expression of USP53, in presence of 2×Taq PCR Master Mix and SYBR Green (SolarBio), with 1 µl cDNA as the template. PCR procedure was set as follows: 95 ℃ for 5 min 10 s, 60 ℃ for 10 s, 72 ℃ for 15 s, followed with 40 cycles of 72 ℃ for 1 min 30 s, 40 ℃ for 1 min, melting 60–94 ℃, every 1 ℃ for 1 s, and finally incubation at 25 ℃ for several min. For detection of one marker, three technical repeats were set for one experiment, and three individual experiments were performed. The data was calculated with 2^−ΔCt^ or 2^−ΔΔCt^ method. The primers were synthesized by General Biol, and the sequences were shown in the following, USP53 forward: 5’-TTATCAGCCTGGAAGTAT-3’; USP53 reverse: 5’-GCATCTCCCTGACAAAC-3’; β-actin forward: 5’- GGCACCCAGCACAATGAA − 3’; β-actin reverse: 5’- TAGAAGCATTTGCGGTGG − 3’.

### Western Blot

Western and IP lysis buffers (Beyotime Institute of Biotechnology, Shanghai, China) supplemented with phenylmethanesulfonyl fluoride (1 mM) were used for protein extraction. After concentration determination, the protein was separated with SDS-PAGE (20 µl protein for one lane), and transferred onto polyvinylidene fluoride membrane (Abcam, Cambridge, UK), which was then blocked for 60 min, and incubated with the following primary antibodies at 4 ℃ overnight in the dark: rabbit anti-USP53 (1:500; cat. No. A14353, Abclonal, Shanghai, China), rabbit anti-ZMYND11 (1:1000; cat. No. GTX103403, GeneTex, Inc., Irvine, CA, USA), and mouse anti-β-actin (1:1000; cat. No. sc-47,778, Santa Cruz Biotechnology Inc. USA). As soon as the membrane was rinsed, goat anti-rabbit or anti-mouse secondary antibodies were incubated at 37 °C for 45 min with HRP (1:5000; Beyotime Institute of Biotechnology), followed by ECL reagent interaction and signal exploration. Gel-Pro-Analyzer software was used to analyze the optical density of the bands. β-actin served as the internal control.

### Immunohistochemistry Staining

The tissues were made into routine paraffin sections, which underwent deparaffination with xylene and ethanol, and antigen retrieval at boiling. Afterwards, 3% H_2_O_2_ was used to eliminate the peroxidase, and 1% BSA was used to block the non-specific antigen. Incubation with primary antibody against Ki-67 (1:50; cat. No. AF0198, Affinity, Cincinnati, OH, USA) was performed at 4 ℃ overnight, and that of secondary antibody labeled with HRP was executed at room temperature for 60 min. Subsequently, the sections interacted with DAB reagent, stained with hematoxylin, soaked with 1% hydrochloric acid/ethanol, dehydrated with ethanol and xylene, finally mounted with gum.

The staining results were scored according to a previous report [24]: percentage of immunoreactive cells: 0 (0–5%), 1 (6–25%), 2 (26–50%), 3 (51–75%) and 4 (76–100%); and staining intensity: 1 (negative), 1 (weak), 2 (moderate) and 3 (intense). USP53 expression was scored by multiplying intensity and percentage. Statistically, a staining score below 6 represents low expression, and a score above 7 represents high expression.

### CCK-8 Assay

In 96-well plates, cells were treated with CCK-8 reagent (10 µl per well) for two hours. After that, the optical density of the supernatant was determined using a microplate reader at 450 nanometers.

### Colony Formation Assay

The MCF-7 and MDA-MB-231 cells with stable expression of USP53 were used for colony formation assay. The cells were seeded in culture dishes with 300 cells in per dish, and culture for about two weeks. The medium was refreshed every three days. After culture for two weeks, the cell clones in dished were fixed and stained with Giemsa stain (Jiancheng Bioengineering Institute, Nanjing, China) for 5 min, and the clone numbers were counted.

### Flow Cytometry

The flow cytometry was performed to detect the cell cycle and apoptosis. The cells were collected and fixed with 70% ethanol at 4 ℃ overnight. The cells were washed with PBS, incubated with RNase A at 37 °C for 30 min, and stained with propidium iodide (PI) in the dark for 30 min. Then the cell cycle phase was determined by flow cytometer (Agilent, Santa Clara, CA, USA). The proliferative index was calculated based on the cell percentage in each phase: (G2 + S)/G1.

For apoptosis detection, the cells were treated with Annexin V-FITC at room temperature for 10 min in the dark, and then stained with PI for 5 min. Immediately, the determination was performed with flow cytometer.

### Measurement of Activity of Caspase-3/9

The activity of caspase-3 and caspase-9 was assessed with Caspase 3 Activity Assay Kit or Caspase 9 Activity Assay Kit (Beyotime Institute of Biotechnology) according to the manufacturer’s protocols. Briefly, the cells were collected and lysed on the ice, and the protein concentration was determined with Bradford Protein Assay Kit (Beyotime Institute of Biotechnology). Afterwards, the sample was incubated with acetyl-Asp-Clu-Val-Asp p-nitroanilide (Ac-DEVD-pNA) substrate at 37 ℃ for 1 h to produce the yellow pNA, and the absorbance at 405 nm was measured with a microplate reader. The standard curve was drawn using standard pNA with grading concentrations, and activity of caspase-3 or caspase 9 was calculated based on the pNA concentration. One unit was the amount of enzyme that will cleave 1.0 nmol of the colorimetric substrate Ac-DEVD-pNA per hour at 37 ℃ under saturated substrate concentrations.

### Reactive Oxygen Species (ROS) Determination

The ROS content in cells were determined with a ROS Assay Kit (Biosharp Life Science, Hefei, Anhui, China) according to the manufacturer’s instruction. The collected cells were firstly incubated with dichlorodihydrofluorescein-diacetate (H2DCFDA) reagent (10 µM) at 37 ℃ for 30 min in the dark. Then the cells were washed twice, and the fluorescence intensity in cells was detected using flow cytometer.

### Mitochondrial Membrane Potential (MMP) Evaluation

The MMP of cells was evaluated using a Mitochondrial Membrane Potential Detection Kit (JC-1) (Biosharp Life Science). The cells cultured on the glass sides were incubated with JC-1 reagent at 37 ℃ for 20 min, and washed by buffer twice. The images were acquired with a fluorescence microscope at 200× magnification. The MMP ratio was calculated by JC-1 aggregates (red)/JC-1 monomers (green).

### Immunofluorescence Staining

Immunofluorescence staining was applied for detection of USP53 and ZMYND11 location in cells. The cells were pre-seeded on glass sides, and fixed with 4% paraformaldehyde (Sinopharm Chemical Reagent Beijing Co., Ltd, Beijing, China) for 15 min, permeated with 0.1% triton-100 (Beyotime Institute of Biotechnology) for 30 min, and blocked with 1% bovine serum albumn (BSA) (Sangon, Shanghai, China) for 15 min. Subsequently, the cells were incubated with antibody against with USP53 (1:100; cat. No. H00054532-B01P) or ZMYND11 (1:100; cat. No. H00054532-B01P, Abnova, Taipei City, Taiwan) at 4 ℃ overnight, and incubated with secondary antibody conjugated with Alexa Fluor^®^ 488 or 555 fluorescent dye (1:200; CST, Boston, MA, USA) for 60 min. Finally, the nuclei were stained with DAPI (Aladdin, Shanghai, China), and sides were mounted with anti-fading reagent (SolarBio). The images were acquired at 400× magnification.

### Co-Immunoprecipitation (Co-IP)

Co-IP was applied with a Co-IP Assay Kit (Pierce, Rockford, IL, USA) to verify the interaction between USP53 and ZMYND11, or ZMYND11 and ubiquitin. The antibody was pre-conjugated onto AminoLink resin, and incubated with cells lysate at room temperature for 2 h. After washing, the antigen-antibody complex was eluted and collected for SDS-PAGE according to the previous description. The antibody against HA (cat. No. AE008, Abclonal) or myc tag (cat. No. AE070, Abclonal) was used at 1:500 dilution.

### Xenograft Model

Healthy female BALB/C nude mice with six-week-old was kept in a controlled environment (12 h light/12 h dark cycles, 21–23 ℃, humidity of 45–55%) with free access to food and water. The MCF-7 cells with USP53-stable-expression or -knockdown were subcutaneously inoculated in mice with 10^7^ cells per mouse (*n* = 6 in each group). The tumor size was measured every three days using a vernier caliper. At 21 days post inoculation, the mice suffered euthanasia of 60% CO_2_ inhalation, and the tumors were isolated for subsequent detections.

The feeding and experiments of animals were carried out according to Guide for the Caree and Use of Laboratory Animals (8th, NIH), and approved by the Ethics Committee of Qinhuangdao First Hospital (approval number: 2022k009).

### TUNEL

The tumor tissue was made into conventional paraffin sections. After deparaffination with xylene and ethanol, the sections were permeated with 0.1% triton X-100, and incubated with TUNEL reagent (Roche, Nutley, NJ, USA) at 37 ℃ for 60 min in a humid and dark box. Then the sections were stained with DAPI, and mounted with anti-fading reagent. The images were acquired at 400× magnification.

### Statistical Analysis

The data in this study were shown as mean ± standard deviation (SD), and analyzed utilizing R or GraphPad Prism software. The data from two independent groups were compared with unpaired t test or Wilcoxon rank sum test (non-normal or heterogeneity of variance). The data from two paired groups were compared with paired t test or Wilcoxon sign rank test (non-normal). The data in multiple groups were analyzed with ANOVA or Kruskal-Wallis (non-normal or heterogeneity of variance), followed with Bonferroni’s multiple comparisons test. The data from immunohistochemistry staining of breast cancer specimens were analyzed with Chi-square or Fisher’s exact tests. A *p* value less than 0.05 was considered as statistically significant.

## Results

### USP53 was Low Expressed in Breast Cancer Tissues

**Fig. 1:**
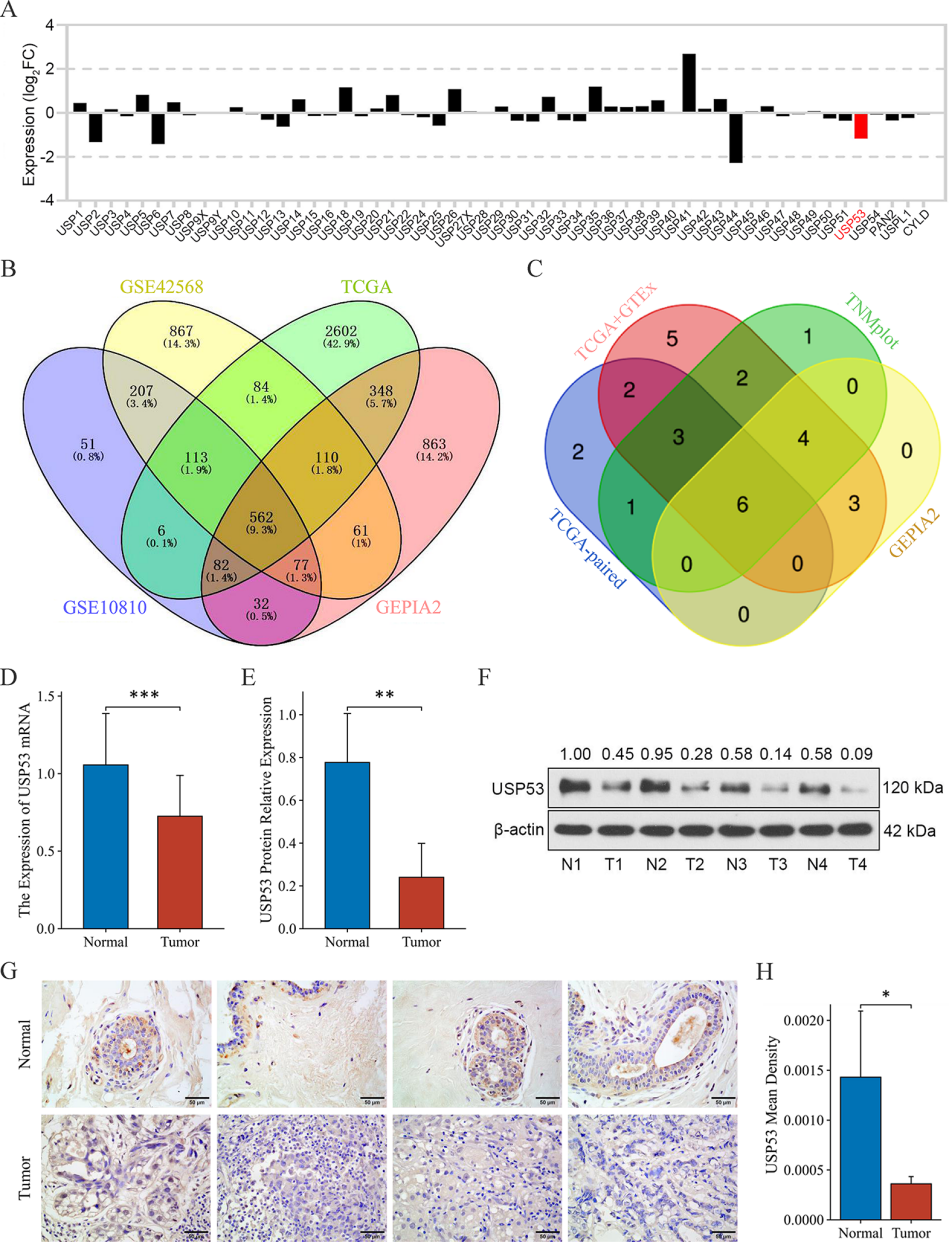
The expression of USP53 was down-regulated in breast cancer tissues. **A** Expression of USP family members in breast cancer from the TCGA database. **B** Intersection of down-regulated genes in breast cancer from GSE10810, GSE42568, TCGA and GEPIA2. **C** USP53 expression in pan-cancer samples from Kaplan-Meier Plotter, GEPIA2, TCGA and GTEx databases. **D** USP53 mRNA levels in 30 pairs of breast cancer and para-carcinoma tissues were detected by real-time PCR. **E**, **F** USP53 protein levels in breast cancer and para-carcinoma tissues were assessed by Western blot. **G**, **H** USP53 expressions in breast cancer and para-carcinoma tissues were determined by immunohistochemical staining. *p < 0.05; **p < 0.01; ***p < 0.001

The differential expression of the USP family in breast cancer compared to normal breast tissue was shown in Fig. 1A, where USP53 expression was down-regulated (log2FC= -1.168, *p* < 0.001). A total of 562 down-regulated genes in breast cancer were screened from the intersection of GSE10810, GSE42568, TCGA and GEPIA2 (Fig. 1B), including only one member of the USP family, USP53 (Supplemental Fig. 1A).

The differences in USP53 expression between pan-cancer and normal tissues in Kaplan-Meier Plotter, GEPIA2, TCGA and GTEx databases were shown in Supplemental Fig. 1B-E. The intersection of statistically significant cancer species in the four analyses contained BRCA, THCA, UCEC, LUSC, LUAD and KIRC, all of which showed down-regulated expression of USP53 compared with normal tissues (Fig. 1C). The full names and abbreviations of the 33 cancers were shown in Tab. S1.

We collected thirty pairs of breast cancer and para-carcinoma tissues, and the real-time PCR result displayed that USP53 was downregulated in breast cancer samples, compared with paired para-carcinoma tissues (Fig. 1D). Similar results was shown in western blot (Fig. 1E and F) and immunohistochemistry (Fig. 1G and H).

#### Clinical Correlation of USP53 with Breast Cancer

**Fig. 2:**
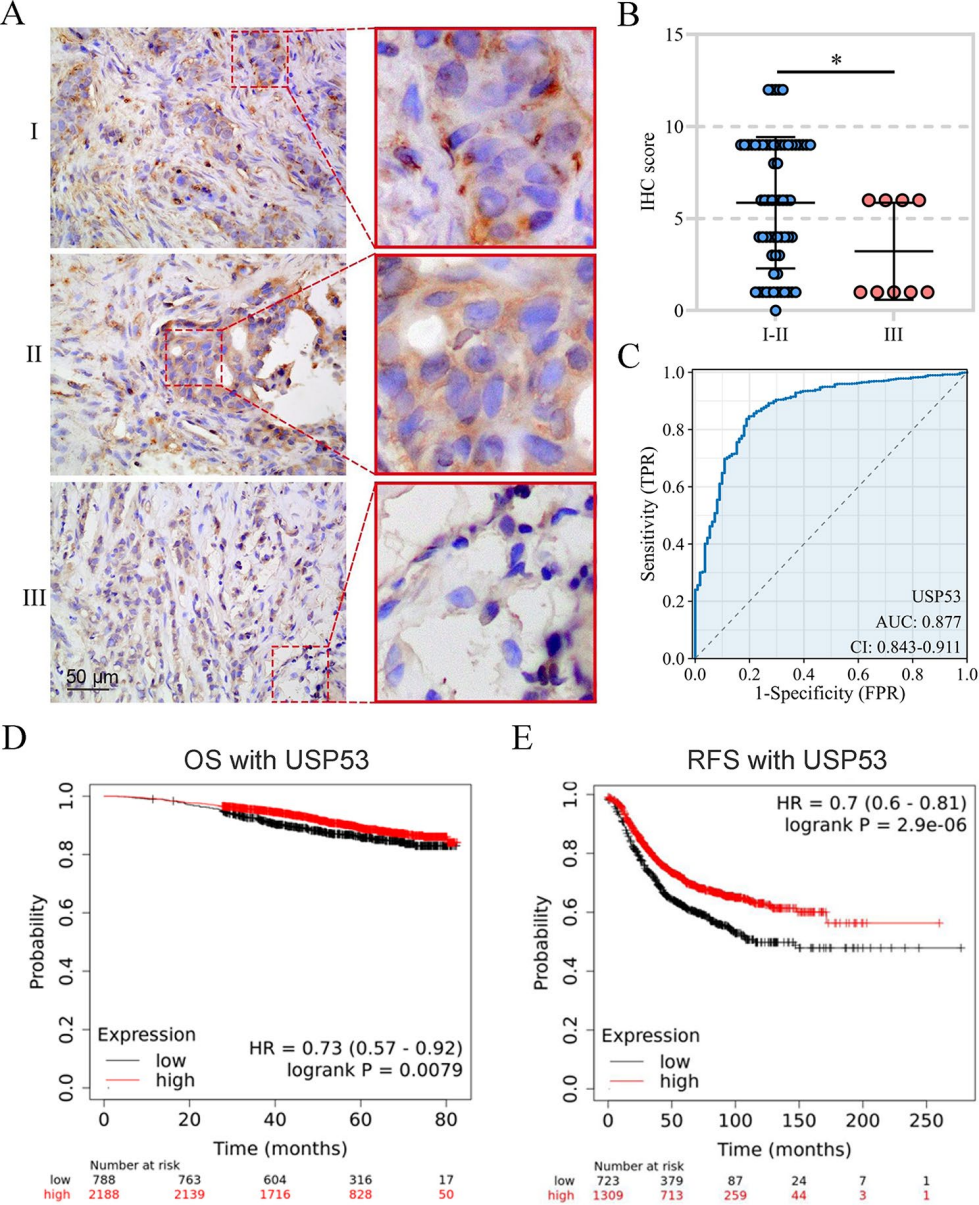
Clinical correlation analysis of USP53 and breast cancer. **A, B** The expression of USP53 in breast cancer tissues of different clinical stages was evaluated by immunohistochemistry. **C** ROC analysis of the diagnostic and predictive value of USP53 in breast cancer. **D** OS curves of breast cancer patients with high and low expression of USP53. **E** RFS curves of breast cancer patients with high and low expression of USP53. *p < 0.05

Additionally, we also collected other 73 breast cancer cases. The analysis from immunohistochemistry staining exhibited that the low expression of USP53 was associated with TNM stage of breast cancers, and more cases with III stage possessed lower USP53 level than those with I/II stages (Table 1; Fig. 2A and B). These results suggested that the decreased USP53 may be involved in development of breast cancer. ROC analysis showed that USP53 had good diagnostic accuracy for breast cancer with an area under curve (AUC) of 0.877 (Fig. 2C). Kaplan-Meier Plotter survival analysis suggested that breast cancer patients with low expression of USP53 had significantly worse overall survival (OS, Fig. 2D) and relapse free survival (RFS, Fig. 2E) than those with high expression.

**Table 1:** The correlation between USP53 and clinicopathologic features in breast cancer patients

Variables	Numbers of patients			*p*
	USP53 high expression		USP53 low expression	
**Age**				
≥ 50	16		35	0.13036
< 50	11		11	
**Tumor size**				
≥ 2 cm	18		36	0.27575
< 2 cm	9		10	
**Grade**				
G1/G2	24		35	0.17984
G3	3		11	
**TNM stage**				
I-II	27		37	0.03698
III	0		9	
**ER**				
negative	1		1	> 0.9999
positive	26		45	
**PR**				
negative	9		20	0.39246
positive	18		26	
**HER2**				
negative	15	32		0.22753
positive	12	14		
**Ki-67**				
< 14%	5	7		0.96784
≥ 14%	22	39		
**Axillary lymph nodes**				
negative	20		28	0.25106
positive	7		18	

### Cell Line Selection and Transfection

**Fig. 3:**
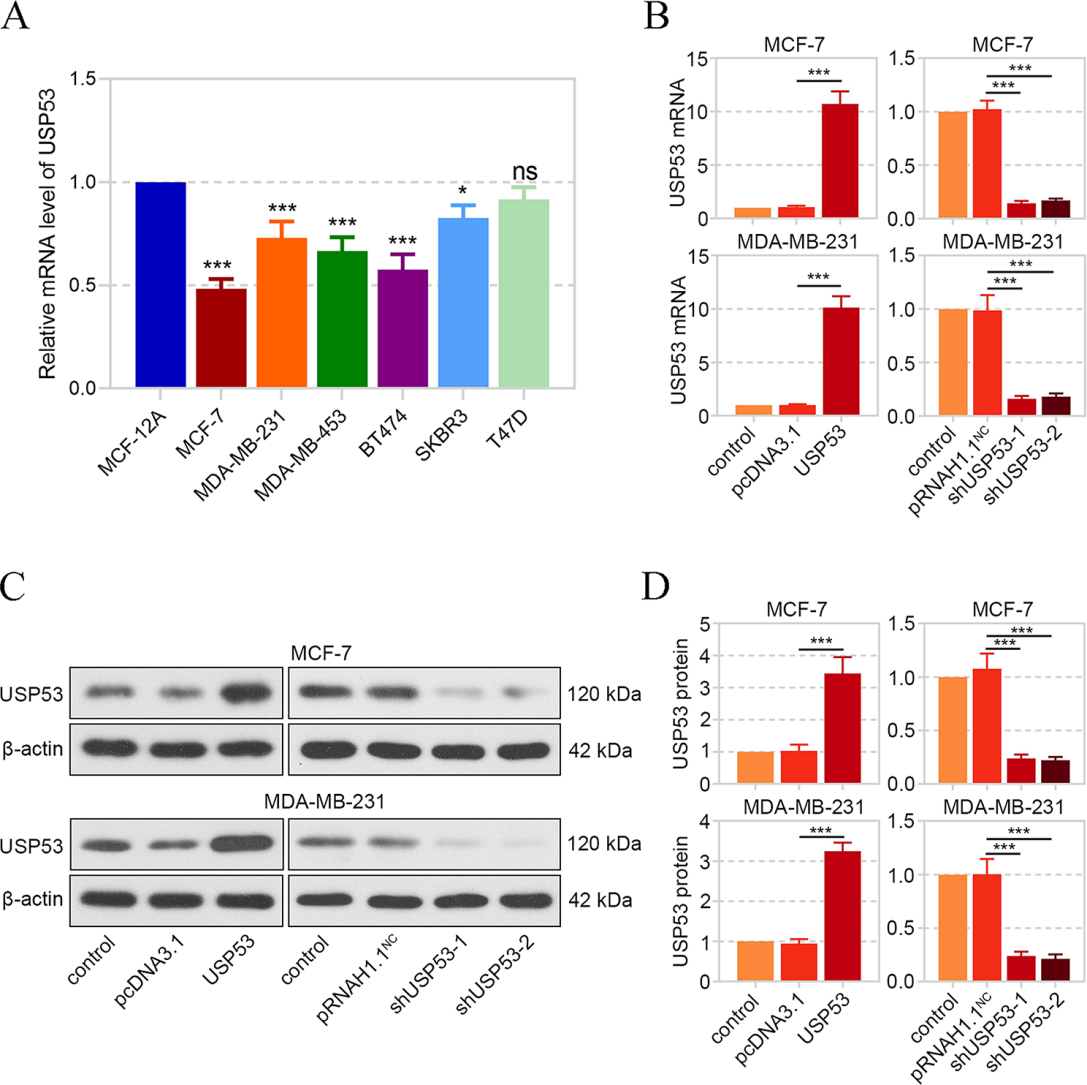
The overexpression and knockdown of USP53 in breast cancer cells. **A** The expression of USP53 mRNA in several breast cancer cell lines was determined by real-time PCR. Subsequently, USP53 coding sequence and the silencing fragment targeting USP53 was inserted into pcDNA3.1 and pRNAH1.1 vectors, respectively, and these vectors were transfected into MCF-7 and MDA-MB-231 breast cancer cells, respectively. **B** USP53 mRNA expression was confirmed by real-time PCR after transfection. **C, D** USP53 protein expression was confirmed by western blot. ***p < 0.001

We detected USP53 mRNA expression levels in human immortalized mammary epithelial cell line MCF-12 A and six breast cancer cell lines (MCF-7, T47D, BT474, SKBR3, MDA-MB-453 and MDA-MB-231), and found that the expression of USP53 in all breast cancer cell lines, except TD47, was significantly lower than that in MCF-12 A cell line (Fig. 3A). Combining the current results with the actual research background, we chose to continue the follow-up experiments employing MCF-7 and MDA-MB-231.

To investigate the function of USP53 in breast cancer, the USP53 overexpression or knockdown vector was transfected into MCF-7 and MDA-MB-231 breast cancer cells, and the expression efficiency was confirmed by real-time PCR (Fig. 3B) and western blot (Fig. 3C andD).

#### USP53 Inhibited the Proliferation and Cell Cycle Transition of Breast Cancer Cells

**Fig. 4:**
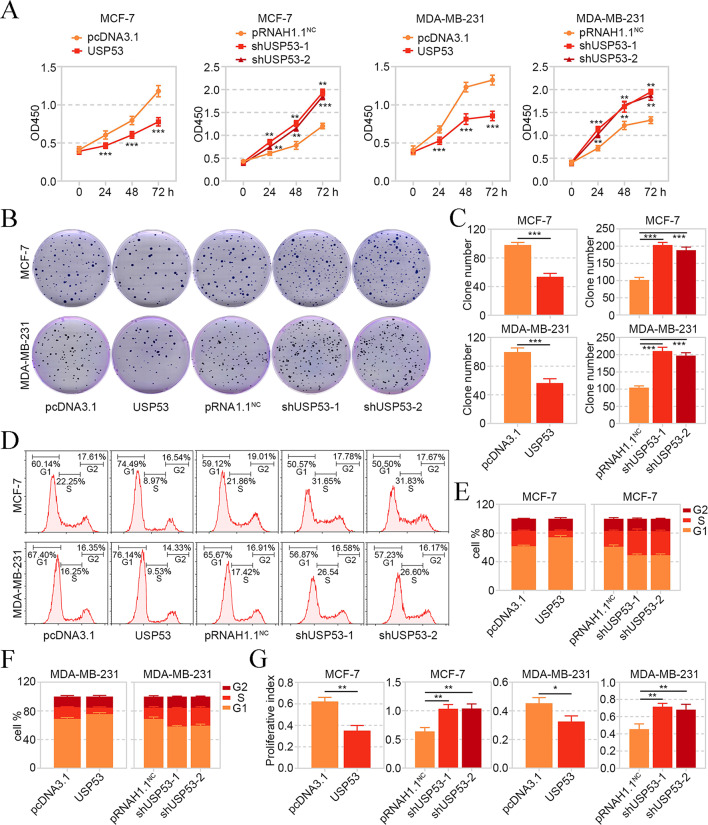
USP53 inhibited the proliferation and cell cycle transition of breast cancer cells. **A** CCK-8 assay was applied to detect the viability of MCF-7 and MDA-MB-231 cells with USP53 overexpression or silencing. **B, C** The colony formation ability of MCF-7 and MDA-MB-231 cells was assessed by the colony formation assay. **D-F** Flow cytometry was performed to determine the cell percentage in each cell cycle phase. **G** The proliferative index was calculated according to the cell percentage in each phase of flow cytometry results by (G2 + S)/G1. *p < 0.05, **p < 0.01, ***p < 0.001

Afterwards, the proliferation of USP53-overexpressed or -silenced MCF-7 and MDA-MB-231 was measured. CCK-8, colony formation assays and flow cytometry revealed that enhanced expression of USP53 suppressed the viability and colony formation, and delayed cell cycle transition of breast cancer cells, while the knockdown of USP53 facilitated cell viability and colony formation, and accelerated cell cycle transition (Fig. 4A-G). The results in this section suggested that USP53 inhibited proliferation of breast cancer cells in vitro.

#### USP53 Induced Apoptosis and Mitochondrial Injury of Breast Cancer Cells

**Fig. 5:**
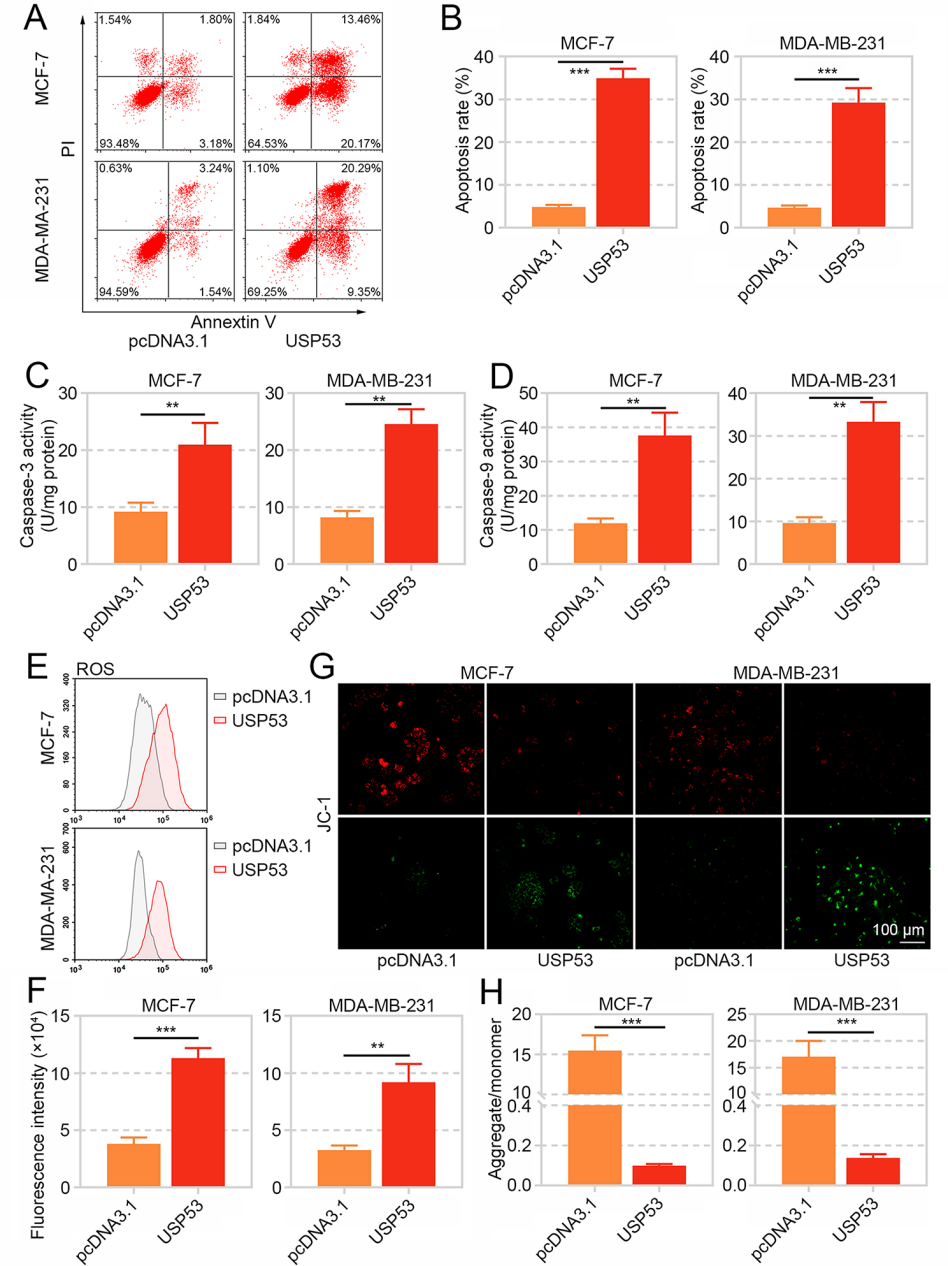
USP53 enhanced apoptosis and mitochondrial injury in breast cancer cells. **A, B** The apoptosis of MCF-7 and MDA-MB-231 cells with after ectoptic expression of USP53was evaluated by Annexin V/PI staining combined with flow cytometry. **C, D** The activities of caspse-3 and caspase-9 in MCF-7 and MDA-MB-231 cells with USP53 overexpression were measured with kits. **E, F** The ROS contents in USP53-overexpressed MCF-7 and MDA-MB-231 cells were examined by H2DCFDA staining and flow cytometry. **G, H** The mitochondria was stained by JC-1 reagent, and the mitochondrial membrane potential was calculated by aggregate (red)/monomer (green). **p < 0.01, ***p < 0.001

Subsequently, flow cytometry data exhibited that USP53 overexpression induced apoptosis of MCF-7 and MDA-MB-231 cells (Fig. 5A and B). Moreover, the activity of apoptosis executors, caspase-3 and caspase-9, was significantly elevated after USP53 overexpression (Fig. 5C andD).

Flow cytometry also revealed that USP53 led to a significant production of reactive oxygen species (ROS) (Fig. 5Eand F). JC-1 staining demonstrated the dramatic reduction of mitochondrial membrane potential in USP53-overexpressed MCF-7 and MDA-MB-231 cells (Fig. 5G and H).

These results revealed that USP53 could induce apoptosis and oxidative stress injury in breast cancer cells.

#### ZMYND11 was Associated with USP53 and Breast Cancer Prognosis

**Fig. 6:**
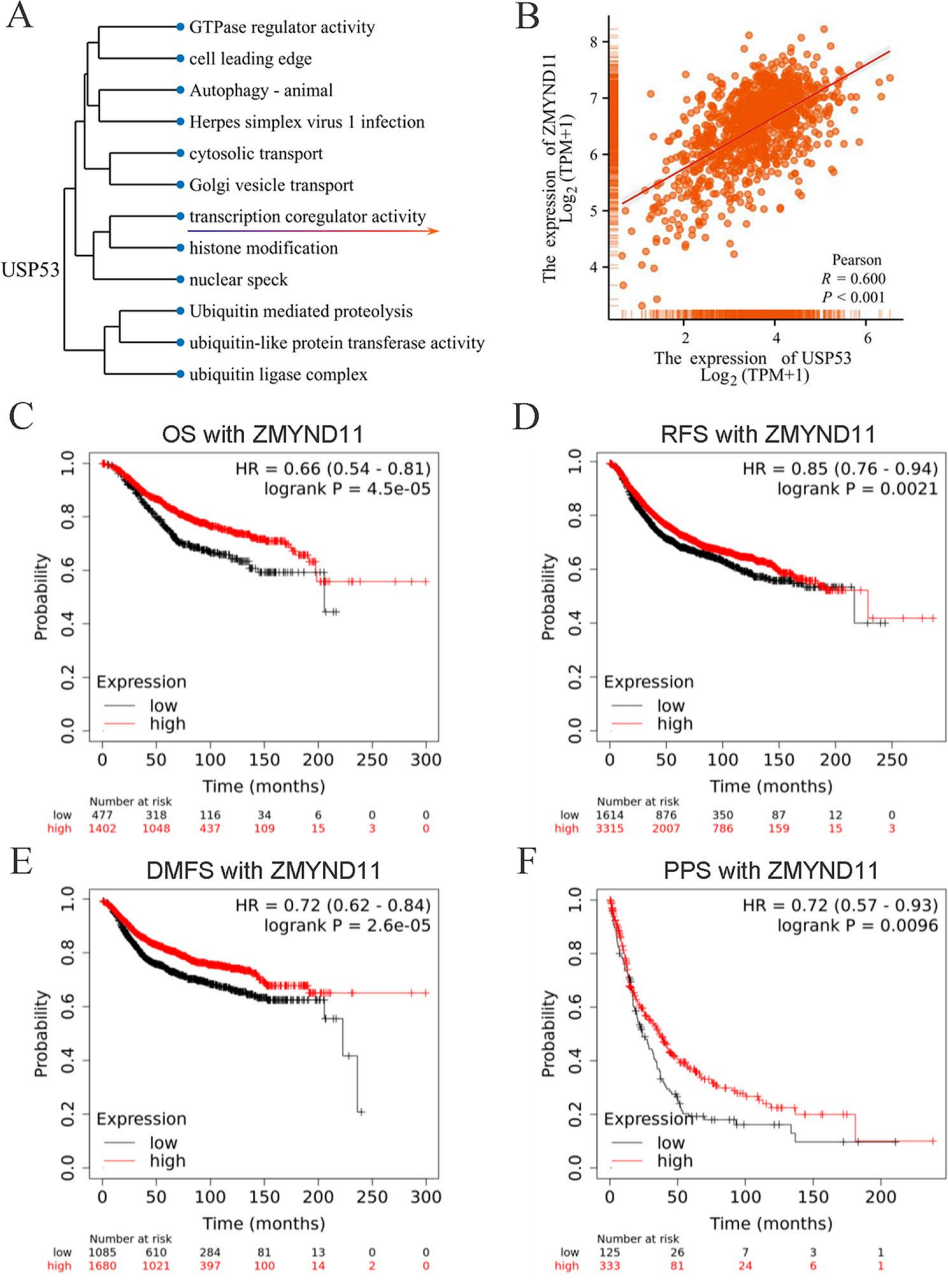
Correlation of ZMYND11 with USP53 and breast cancer prognosis. **A, B** Functional cluster analysis of USP53 in breast cancer and its correlation with ZMYND11. **C** OS curves of breast cancer patients with high and low expression of ZMYND11. **D** RFS curves of breast cancer patients with high and low expression of ZMYND11. **E** DMFS curves of breast cancer patients with high and low expression of ZMYND11. **F** PPS curves of breast cancer patients with high and low expression of ZMYND11

A total of 1785 USP53-correlated genes were obtained by single gene correlation screening of breast cancer samples from TCGA database. Then, the functional enrichment analysis and cluster analysis of this gene set showed that USP53 was correlated with the activity of transcriptional coregulators, among which it was positively correlated with ZMYND11 (Fig. 6A and B). Kaplan-Meier Plotter survival analysis showed that breast cancer patients with low expression of ZMYND11 had worse OS (Fig. 6C), RFS (Fig. 6D), distant metastasis-free survival (DMFS, Fig. 6E) and post-progression survival (PPS, Fig. 6F) than those with high expression.

### USP53 Interacted with ZMYND11 and Blocked its Ubiquitination

**Fig. 7:**
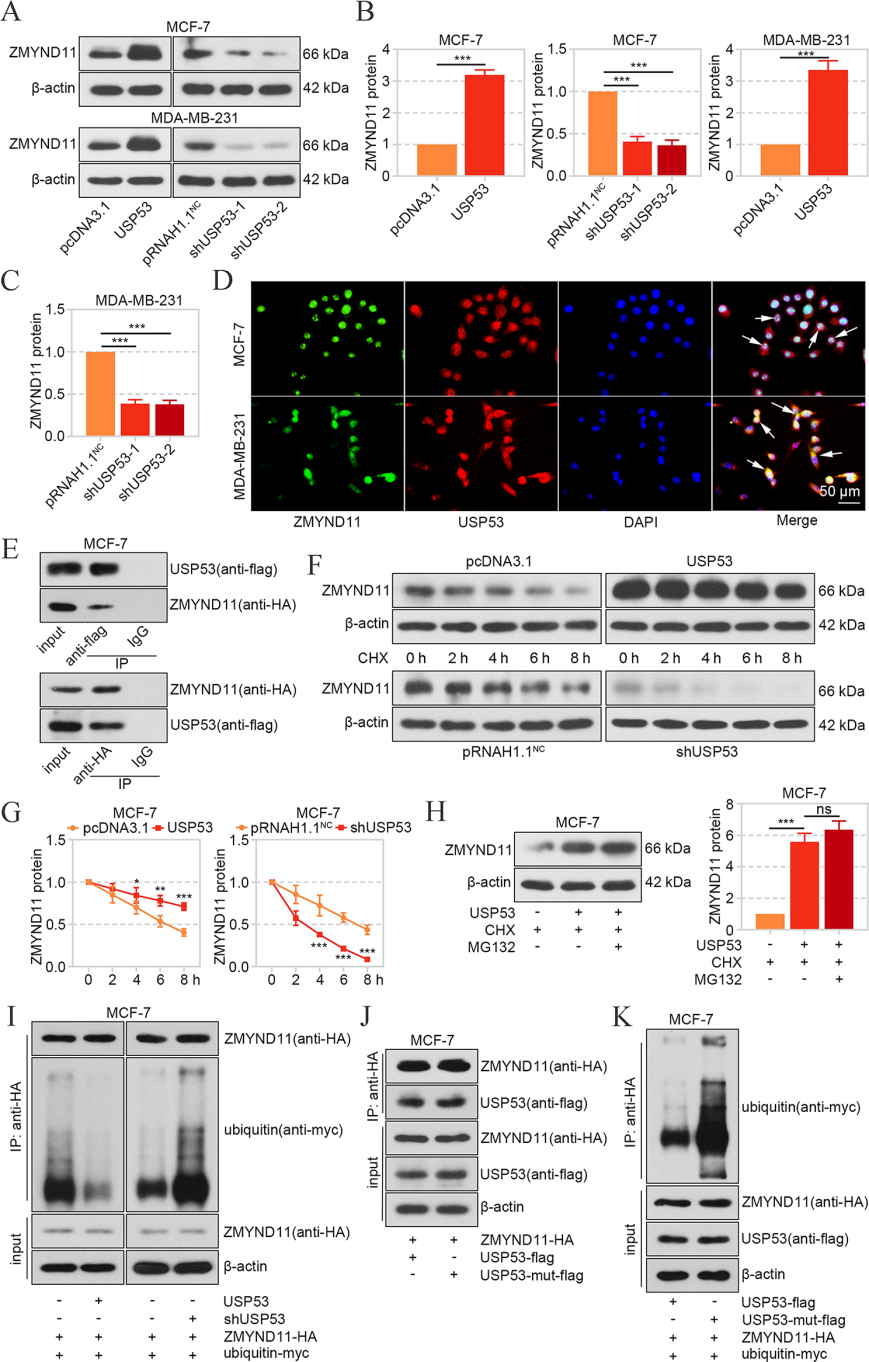
USP53 interacted with ZMYND11 and blocked its ubiquitination. **A-C** The expression of ZMYND11 in MCF-7 and MDA-MB-231 cells after overexpression or knockdown of USP53 was determined by western blot. **D** The co-localization of USP53 and ZMYND11 was verified in MCF-7 and MDA-MB-231 cells. **E** The binding between USP53 and ZMYND11 was confirmed in MCF-7 cells with transfection of USP53-flag and ZMYND11-HA vectors. **F, G** The ZMYND11 protein levels were determined in MCF-7 cells after CHX treatment for different hours, and the ZMYND11 degradation rate was assessed. **H** The ZMYND11 protein level in MCF-7 cells with USP53 overexpression, combined with CHX and MG132 administration for 8 h. **I** The ubiquitinated level of ZMYND11 in MCF-7 with USP53 overexpression or knockdown was measured by IP. **J** The mutated USP53 with deficiency of 33–50 amino acid residues was constructed and transfected into MCF-7 cells, and the binding between ZMYND11 and wild type or mutated USP53 was verified. **K** The ubiquitination of ZMYND11 was measured with IP after transfection of wild type or mutated USP53 overexpression vector, and was calculated by aggregate (red)/monomer (green). *p < 0.05; **p < 0.01; ***p < 0.001; ^ns^No significance

Given the potential interaction between USP53 and ZMYND11, we determined the ZMYND11 level after USP53 changes. Western blot results showed that the ZMYND11 protein level was increased after USP53 overexpression and decreased after USP53 silencing in MCF-7 and MDA-MB-231 cells (Fig. 7A-C). The immunofluorescent staining results showed the co-localization of USP53 and ZMYND11 (Fig. 7D), and Co-IP demonstrated their binding in breast cancer cells (Fig. 7E). Subsequently, CHX was used to block the protein translation, and western blot results demonstrated that USP53 delayed the degradation of ZMYND11, while USP53 knockdown accelerated its degradation (Fig. 7F and G). The application of proteasome inhibitor MG132 did not exacerbate the effect of USP53, suggesting that USP53 blocked ZMYND11 degradation via proteasome (Fig. 7H). Considering that USP53 was reported to act as a DUB, the ubiquitination of ZMYND11 was assessed. As was shown in Fig. 7I, the ubiquitination of ZMYND11 was attenuated by USP53 overexpression, while aggravated by USP53 knockdown. Cys-box of USP53 (33–50 amino acid residues) was reported to be essential for catalytic properties [25], so the mutated USP53 plasmid with deficiency of 33–50 animo acid residues was constructed. The Co-IP results revealed that the deficiency mutation neither affected the expression of USP53 nor its binding with ZMYND11, but deprived USP53 of DUB activity (Fig. 7J and K). These results suggested that USP53 interacted with ZMYND11 and intercepted its ubiquitination in breast cancer cells.

### USP53 Functioned by Regulating ZMYND11 Expression

**Fig. 8:**
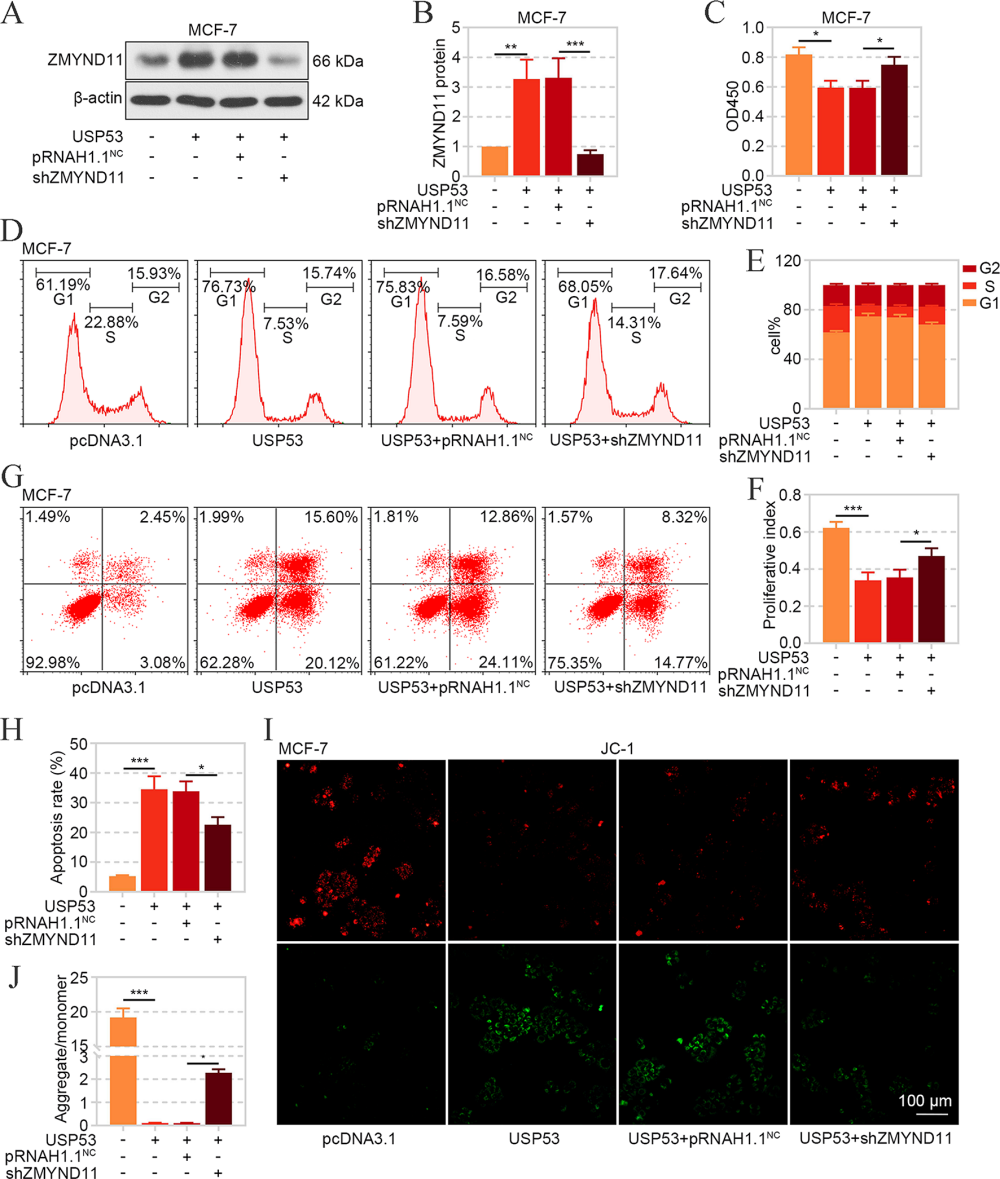
The effects of USP53 on breast cancer cells was abolished by ZMYND11 knockdown. **A, B** The protein level of ZMYND11 after USP53 overexpression or/and ZMYND11 silencing was determined by western blot. **C** The viability of MCF-7 cells with USP53 overexpression or/and ZMYND11 silencing was assessed by CCK-8 assay. **D-F** The cell cycle distribution of MCF-7 cells in each phase was detected by flow cytometry and the proliferative index was calculated. **G, H** The apoptosis of MCF-7 with USP53 overexpression or/and ZMYND11 silencing was examined by Annexin V/PI staining and flow cytometry. **I-J** The mitochondrial membrane potential of MCF7 cells with transfection of USP53 overexpression plasmid or/and ZMYND11 knockdown vector was determined by JC-1 staining. *p < 0.05, **p < 0.01, ***p < 0.001

Next, the ZMYND11 silencing vector was transfected into USP53-overexpressed MCF-7 cells, and the results demonstrated that USP53-reduced ZMYND11 level was recovered by its knockdown (Fig. 8A and B). CCK-8 assay, flow cytometry and JC-1 staining exhibited that USP53-induced viability decline, cell cycle arrest, apoptosis and mitochondrial injury were abrogated by ZMYND11 silencing to different degrees (Fig. 8C-J). These results suggested that USP53 exerted function by positively regulating ZMYND11 expression.

## USP53 Restrained the Growth of Breast Cancer Cells in Nude Mice

**Fig. 9:**
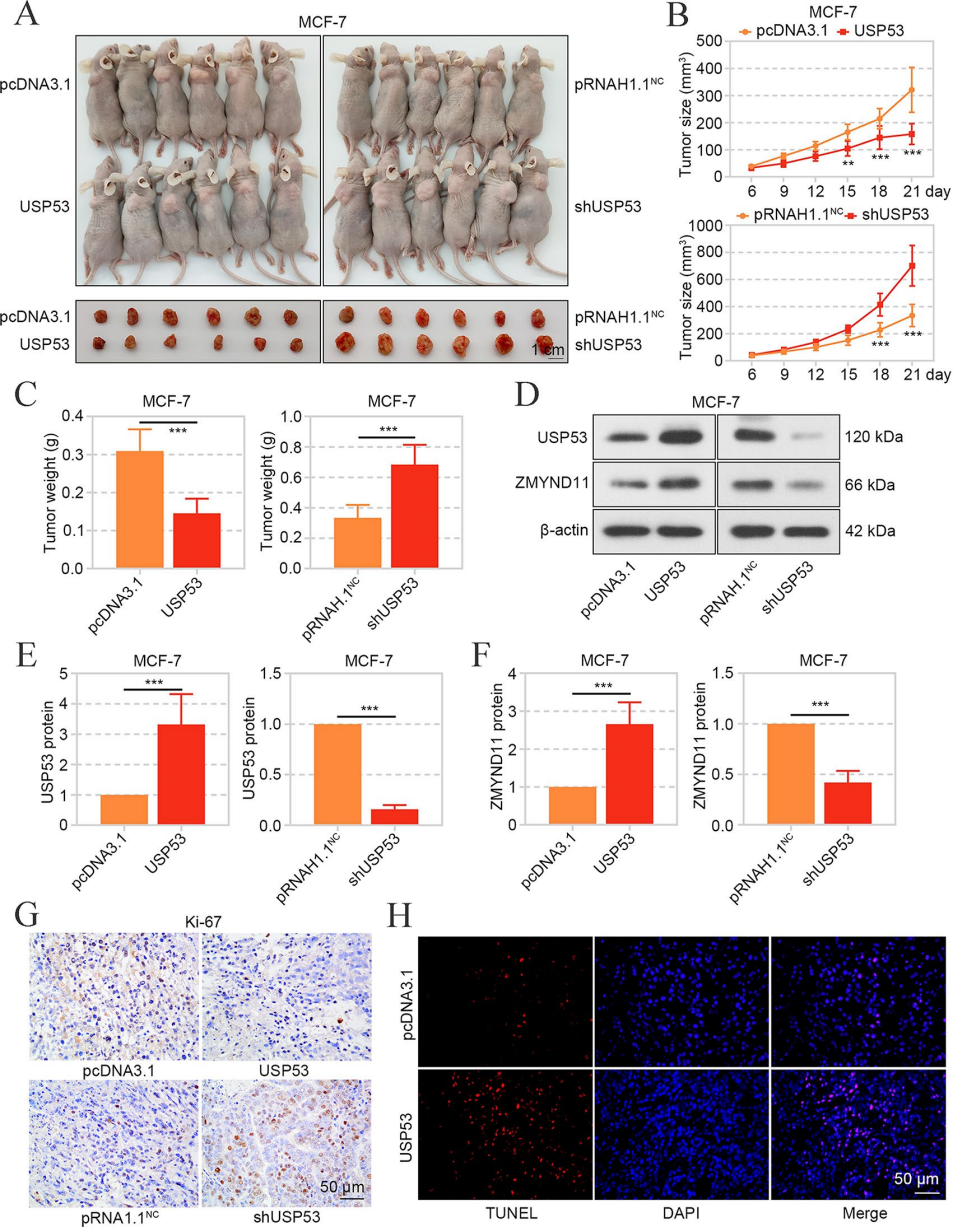
USP53 restrained the growth of breast cancer cells in nude mice. **A** The MCF-7 cells with stable expression or knockdown of USP53 was subcutaneously inoculated in mice with 10^7^ cells per mouse, and the tumors were isolated 21 days later. The mice and tumors were shown. **B** The tumor size in mice at different times post inoculation. **C** The tumor weight was measured at 21 days post inoculationa. **D-F** The expression of USP53 and ZMYND11 in tumors was detected by western blot. **G** The expression and distribution of Ki-67 in tumors were determined by immunohistochemistry staining. **H** TUNEL was applied to measure apoptosis in tumors. **p < 0.01, ***p < 0.001

Finally, the effects of USP53 on breast cancer was evaluated in vivo. As shown in Fig. 9A-C, the USP53-overexpressed MCF-7 cells grew significantly slow, while USP53-silenced cells grew faster. In the tumors, USP53 promoted the expression of ZMYND11 (Fig. 9D-F), consistent with results in vitro. Moreover, USP53 suppressed the expression of proliferation marker Ki-67, and induced apoptosis in tumors (Fig. 9G and H). These results suggested that USP53 restrained the growth of breast cancer cells in vivo.

## Discussion

The dynamic balance between ubiquitination and deubiquitination is an important mechanism to maintain normal physiological activities of the human body, but some genetic or epigenetic changes may lead to the imbalance of the two, which is closely related to many diseases, including malignant tumors [26–28]. The role of DUBs in cancer is therefore receiving increasing attention from researchers [5]. The involvement of USPs, the biggest family of DUBs, in tumor development has been reported for several decades, and they functioned as tumor-suppressors or oncogenes via catalyzing different substrates [7]. For instance, USP1 stabilized estrogen receptor alpha (ERα) to promote proliferation and invasion of breast cancer cells [29]. USP13 suppressed tumorigenesis through deubiquitination and stabilization of phosphatase and tensin homolog (PTEN) in breast cancer [30]. We used the TCGA database to analyze the expression of USP family members, and found that USP2, USP6, USP44 and USP53 in breast cancer tissues were significantly down-regulated compared with those in normal breast tissues, among which the first three have been studied in this field [31–33]. Subsequently, we screened the gene sets of GSE10810, GSE42568, TCGA and GEPIA2 that are significantly down-regulated in breast cancer and whose cross-sets contain only one member of the USP family, USP53. Further, GEPIA2, Kaplan-Meier Plotter, TCGA and GTEx databases were used to jointly analyze the expression of USP53 in pancarcinoma and corresponding normal tissues, and the cancer types with statistical differences in the four analyses were selected, including breast cancer with low expression of USP53. In order to verify such expression trend, we collected clinical specimens for analysis, and found that both mRNA and protein levels of USP53 were significantly lower in breast cancer tissues than in normal breast tissues. This was consistent with the expression trend of USP53 in lung cancer [14], renal clear cell carcinoma [15], hepatocellular carcinoma [16] and esophageal carcinoma [17], and it acted as a tumor suppressor in these cancers, so we speculated that USP53 should play the similar role in breast cancer.

Next, we used clinical tissue samples to analyze the correlation between USP53 expression and clinicopathological characteristics of breast cancer, and found that the level of USP53 was negatively correlated with the clinical stage of breast cancer, which was consistent with the results of the clinical correlation analysis of USP53 in the study of esophageal cancer by Cheng et al. [17]. ROC analysis showed that USP53 had good diagnostic and predictive value in breast cancer. Kaplan-Meier Plotter survival analysis suggested that USP53 was a prognostic protective factor for breast cancer, and patients with high expression of USP53 could obtain better OS and RFS. In studies of lung cancer, renal clear cell carcinoma, hepatocellular carcinoma and esophageal cancer, patients with high expression of USP53 also showed better survival outcomes, suggesting that USP53 may be a broad-spectrum prognostic protective factor for cancer [14–17].

To confirm the inhibitory effect of USP53 on breast cancer, we conducted cell function experiments. First, the down-regulation trend of USP53 in six breast cancer cell lines was verified, and stable cell lines (MCF-7 and MDA-MB-231) with USP53 overexpression or knockdown were successfully constructed for follow-up experiments. Through CCK-8, clonogenesis assay and flow cytometry, it was confirmed that USP53 can reduce DNA synthesis by triggering G1 phase arrest, thus inhibiting the proliferation activity and clonogenesis ability of breast cancer cells. In similar studies, USP53 was also shown to have proliferative inhibitory effects on lung cancer, renal clear cell carcinoma, hepatocellular carcinoma and esophageal carcinoma [14–17]. Caspase-3 is one of the most important enzymes in the process of apoptosis, which can activate Procaspase-9, and Caspase-9 further activates Caspase-3, forming a positive feedback loop and ultimately mediating apoptosis [34–36]. In our study, USP53 overexpression significantly increased the activities of Caspase-3 and Caspase-9, thereby effectively increasing the apoptosis rate of breast cancer cells. Consistent with our results, overexpression of USP53 had significant pro-apoptotic effects in the studies of lung cancer, hepatocellular carcinoma and esophageal cancer [14, 16, 17]. Moreover, USP53 caused mitochondrial injury, and we assumed that mitochondrial damage may mediated USP53-induced cell apoptosis. Mitochondria are the energy production structure of cells and the main sites of aerobic metabolism, and normal transmembrane potential is the prerequisite for maintaining oxidative phosphorylation and ATP synthesis [37]. The sharp increase of ROS content can induce the increase of mitochondrial inner membrane permeability, which can decrease or disappear the transmembrane potential difference, and then cause cell damage [38]. Disrupting the normal mitochondrial metabolism and inducing excess ROS production could be considered as novel anti-cancer approaches. An inhibitor of mitochondrial TCA cycle, CPI-613, promoted ROS-mediated apoptosis in pancreatic cancer cells [39]. A microtubule-targeting agent CYT997 induced apoptosis through triggering mitochondrial ROS generation in gastric cancer cells [40]. Our data showed that USP53 induced mitochondrial injury and elevated ROS production, which may mediate the apoptosis and proliferation inhibition in USP53-overexpressed breast cancer cells. Cheng et al. reported that USP53 also caused the same effect of mitochondrial damage in esophageal cancer cells [17].

A catalytic triad consisting of cysteine, histidine and aspartic acid exists in most members of the USPs [10]. In response to diubiquitin binding, the catalytic cysteine residue deprotonates, attacking the isopeptide linkage, which forms a first, negatively charged tetrahedral intermediate stabilized by an oxyanion hole in the catalytic domain. The proximal ubiquitin is is released later, followed by the formation of an acyl intermediate. Another tetrahedral intermediate results from the following deacylation reaction carried out by a water molecule. When this intermediate collapses, the distal ubiquitin is released, allowing the apoenzyme to regenerate [6, 41]. Currently, USP53 has been shown to catalyze deubiquitination of several proteins and prevent their degradation, such as FKBP51, IκBα and cytochrome C [14–16]. Bioinformatics analysis showed a positive correlation between the expression of USP53 and ZMYND11 in breast cancer tissues. Survival analysis found that breast cancer patients with high expression of ZMYND11 showed better prognosis, which was consistent with the analysis results of Wen et al. [23]. These findings suggest that there may be synergies between ZMYND11 and USP53, and that ZMYND11 may mediate the function of USP53.

Therefore, we conducted a series of experiments focusing on the relationship between USP53 and ZMYND11. Immunofluorescence staining showed that USP53 and ZMYND11 were co-localized in breast cancer cells, and Co-IP assay showed that the two could bind to each other, and both could still bind after the mutation of the active center of USP53, accompanied by the increase of the ubiquitination level of ZMYND11. USP53 is a classical cysteine proteases, and its 33–50 amino acid is identified as catalytic Cys-box [25]. In our results, the absence of this Cys-box only caused USP53 to lose its deubiquitination effect on ZMYND11, but did not affect their binding. This was consistent with a previous paper [16], in which the deletion of 33–50 amino acid only abrogated the deubiquitinaiton of USP53 on cytochrome C in hepatoma cells, and did not affect the binding of the two. These results indicated that Cys-box was not necessary for the binding of USP53 to its substrates, and there may be other subbinding sites. Next, we confirmed by Western blot that overexpression of USP53 could up-regulate the expression of ZMYND11, accompanied by a decrease in its ubiquitination level, while knockdown of USP53 showed an opposite trend. Using CHX to inhibit protein synthesis in breast cancer cells, it was found that overexpression of USP53 delayed the degradation of ZMYND11, while knockdown of USP53 accelerated its degradation. When MG132 was further used to block the ubiquitin-proteasome system (UPS), the expression of ZMYND11 was not further upregulated, which confirmed the degradation of ZMYND11 by UPS pathway, indicating that USP53 effectively stabilized the protein level of ZMYND11 through deubiquitination. This regulatory trend was consistent with the deubiquitination of cytochrome C, IκBα, and FKBP51 by USP53 in hepatocellular carcinoma, renal clear cell carcinoma and lung cancer studies [14–16].

In the subsequent Rescue experiment, we knocked down ZMYND11 on the basis of USP53 overexpression. The results showed that shZMYND11 significantly improved the survival ability of breast cancer cells, promoted the cell cycle process, reduced the number of apoptotic cells, and replicated the mitochondrial transmembrane potential. This indicated that ZMYND11 knockdown reversed the effects of proliferation inhibition, G1 phase arrest, pro-apoptosis and mitochondrial damage in breast cancer cells caused by overexpression of USP53, thereby reverently confirming the in vitro inhibitory effect of USP53 deubiquitination stabilizing ZMYND11 expression. This was consistent with the results of in vitro studies of ZMYND11 in brain glioma and kidney cancer [42, 43]. Finally, we further studied the role and mechanism of USP53 in vivo by constructing the transplanted tumor model of MCF-7 cell nude mice. The results showed that USP53 could significantly inhibit the growth of breast cancer grafts, decrease the proliferation activity of cancer cells, increase the number of apoptotic cells in the tumor, and still regulate the expression level of ZMYND11. The above results are consistent with the results published by YANG et al. that ZMYND11 inhibits GBM in vitro and in vivo [21].

Breast cancer is a common female tumor, contains four subtypes: Luminal A, Luminal B, HER2+++ and triple negative breast cancer [44]. Our data demonstrated that USP53 was downregulated in breast cancer specimens, including the above four subtypes. The cell lines used in our study, MCF-7 and MDA-MB-231, were originated from Luminal A and triple negative breast cancer patients, respectively. Our results demonstrated that USP53 inhibited proliferation and induced apoptosis of MCF-7 and MDA-MB-231 cells in vivo and in vitro, suggesting that the anti-tumor effect of USP53 was extensive in different subtypes of breast cancer. The previous article reported the tumor-suppressive role of ZMYND11 in MDA-MB-231 cells, and our data supplemented its role in MCF-7 cells. These evidences revealed that the anti-cancer function of USP53 may be mediated by ZMYND11 in breast cancer. However, whether this hypothesis holds in other tumors needs to be verified.

## Conclusions

USP53 was low expressed in breast cancer tissues, and its expression was negatively correlated with the clinical stage of breast cancer. USP53 suppressed proliferation and led to apoptosis of breast cancer cells in vitro and in vivo. USP53 induced deubiquitination and stabilization of ZMYND11, both of which were prognostic protective factors for breast cancer. ZMYND11 mediated the function of USP53 in breast cancer progression. These findings may provide new insights into the clinical diagnosis, treatment and prognosis of breast cancer.

### Electronic Supplementary Material

Below is the link to the electronic supplementary material.

Supplementary Material 1

Supplementary Material 2

## Data Availability

No datasets were generated or analysed during the current study.

## Abbreviations

USP53: Ubiquitin-specific peptidase 53
ZMYND11: Zinc finger MYND-type containing 11
DUB: Deubiquitinase
USPs: Ubiquitin-specific peptidases
ROS: Reactive oxygen species
MMP: Mitochondrial membrane potential
Co-IP: Co-immunoprecipitation
AUC: Area under curve
OS: Overall survival
RFS: Relapse free survival
DMFS: Distant metastasis-free survival
PPS: Post-progression survival
PTEN: Phosphatase and tensin homolog
UPS: Ubiquitin-proteasome system

## References

[1] Sung H, Ferlay J, Siegel RL, et al. Global Cancer statistics 2020: GLOBOCAN estimates of incidence and Mortality Worldwide for 36 cancers in 185 countries. CA Cancer J Clin. 2021;71:209–49. 10.3322/caac.21660. 33538338 10.3322/caac.21660

[2] DeSantis CE, Ma J, Gaudet MM, et al. Breast cancer statistics, 2019. CA Cancer J Clin. 2019;69:438–51. 10.3322/caac.21583. 31577379 10.3322/caac.21583

[3] Lee JM, Hammaren HM, Savitski MM, Baek SH. Control of protein stability by post-translational modifications. Nat Commun. 2023;14:201. 10.1038/s41467-023-35795-8. 36639369 10.1038/s41467-023-35795-8 PMC9839724

[4] Culver JA, Li X, Jordan M, Mariappan M. A second chance for protein targeting/folding: Ubiquitination and deubiquitination of nascent proteins. BioEssays. 2022;44:e2200014. 10.1002/bies.202200014. 35357021 10.1002/bies.202200014 PMC9133216

[5] Li Y, Reverter D. Molecular mechanisms of DUBs Regulation in Signaling and Disease. Int J Mol Sci. 2021;22. 10.3390/ijms22030986. 10.3390/ijms22030986 PMC7863924 33498168

[6] Chen S, Liu Y, Zhou H. Advances in the Development Ubiquitin-Specific Peptidase (USP) inhibitors. Int J Mol Sci. 2021;22. 10.3390/ijms22094546. 10.3390/ijms22094546 PMC8123678 33925279

[7] Cruz L, Soares P, Correia M. Ubiquitin-specific proteases: players in Cancer Cellular processes. Pharmaceuticals (Basel). 2021;14. 10.3390/ph14090848. 10.3390/ph14090848 PMC8469789 34577547

[8] Snyder NA, Silva GM. Deubiquitinating enzymes (DUBs): regulation, homeostasis, and oxidative stress response. J Biol Chem. 2021;297:101077. 10.1016/j.jbc.2021.101077. 34391779 10.1016/j.jbc.2021.101077 PMC8424594

[9] Young MJ, Hsu KC, Lin TE, Chang WC, Hung JJ. The role of ubiquitin-specific peptidases in cancer progression. J Biomed Sci. 2019;26:42. 10.1186/s12929-019-0522-0. 31133011 10.1186/s12929-019-0522-0 PMC6537419

[10] Quesada V, Diaz-Perales A, Gutierrez-Fernandez A, Garabaya C, Cal S, Lopez-Otin C. Cloning and enzymatic analysis of 22 novel human ubiquitin-specific proteases. Biochem Biophys Res Commun. 2004;314:54–62. 10.1016/j.bbrc.2003.12.050. 14715245 10.1016/j.bbrc.2003.12.050

[11] Maddirevula S, Alhebbi H, Alqahtani A, et al. Identification of novel loci for pediatric cholestatic liver disease defined by KIF12, PPM1F, USP53, LSR, and WDR83OS pathogenic variants. Genet Med. 2019;21:1164–72. 10.1038/s41436-018-0288-x. 30250217 10.1038/s41436-018-0288-x

[12] Hariri H, Kose O, Bezdjian A, Daniel SJ, St-Arnaud R. USP53 regulates Bone Homeostasis by Controlling Rankl expression in Osteoblasts and bone marrow adipocytes. J Bone Min Res. 2023;38:578–96. 10.1002/jbmr.4778. 10.1002/jbmr.4778 36726200

[13] Kazmierczak M, Harris SL, Kazmierczak P, et al. Progressive hearing loss in mice carrying a mutation in Usp53. J Neurosci. 2015;35:15582–98. 10.1523/JNEUROSCI.1965-15.2015. 26609154 10.1523/JNEUROSCI.1965-15.2015 PMC4659823

[14] Zhao X, Wu X, Wang H, Yu H, Wang J. USP53 promotes apoptosis and inhibits glycolysis in lung adenocarcinoma through FKBP51-AKT1 signaling. Mol Carcinog. 2020;59:1000–11. 10.1002/mc.23230. 32511815 10.1002/mc.23230

[15] Gui D, Dong Z, Peng W, et al. Ubiquitin-specific peptidase 53 inhibits the occurrence and development of clear cell renal cell carcinoma through NF-kappaB pathway inactivation. Cancer Med. 2021;10:3674–88. 10.1002/cam4.3911. 33973730 10.1002/cam4.3911 PMC8178486

[16] Yao Y, Ma W, Guo Y, et al. USP53 plays an antitumor role in hepatocellular carcinoma through deubiquitination of cytochrome c. Oncogenesis. 2022;11:31. 10.1038/s41389-022-00404-8. 35654790 10.1038/s41389-022-00404-8 PMC9163188

[17] Cheng W, Tang Y, Tong X, et al. USP53 activated by H3K27 acetylation regulates cell viability, apoptosis and metabolism in esophageal carcinoma via the AMPK signaling pathway. Carcinogenesis. 2022;43:349–59. 10.1093/carcin/bgab123. 34919659 10.1093/carcin/bgab123

[18] Oates S, Absoud M, Goyal S, et al. ZMYND11 variants are a novel cause of centrotemporal and generalised epilepsies with neurodevelopmental disorder. Clin Genet. 2021;100:412–29. 10.1111/cge.14023. 34216016 10.1111/cge.14023

[19] Hao J, Shen R, Li Y, Zhang Y, Yin Y. Cancer-testis antigen HCA587/MAGE-C2 interacts with BS69 and promotes its degradation in the ubiquitin-proteasome pathway. Biochem Biophys Res Commun. 2014;449:386–91. 10.1016/j.bbrc.2014.05.078. 24866244 10.1016/j.bbrc.2014.05.078

[20] Wei G, Schaffner AE, Baker KM, Mansky KC, Ostrowski MC. Ets-2 interacts with co-repressor BS69 to repress target gene expression. Anticancer Res. 2003;23:2173–8. http://www.ncbi.nlm.nih.gov/pubmed/12894593. 12894593

[21] Yang H, Zhang C, Zhao X, et al. Analysis of copy number variations of BS69 in multiple types of hematological malignancies. Ann Hematol. 2010;89:959–64. 10.1007/s00277-010-0966-5. 20425112 10.1007/s00277-010-0966-5

[22] Yang JP, Yang JK, Li C, et al. Downregulation of ZMYND11 induced by miR-196a-5p promotes the progression and growth of GBM. Biochem Biophys Res Commun. 2017;494:674–80. 10.1016/j.bbrc.2017.10.098. 29066350 10.1016/j.bbrc.2017.10.098

[23] Wen H, Li Y, Xi Y, et al. ZMYND11 links histone H3.3K36me3 to transcription elongation and tumour suppression. Nature. 2014;508:263–8. 10.1038/nature13045. 24590075 10.1038/nature13045 PMC4142212

[24] Jin X, Wang D, Lei M, et al. TPI1 activates the PI3K/AKT/mTOR signaling pathway to induce breast cancer progression by stabilizing CDCA5. J Transl Med. 2022;20:191. 10.1186/s12967-022-03370-2. 35509067 10.1186/s12967-022-03370-2 PMC9066866

[25] Quesada V, Díaz-Perales A, Gutiérrez-Fernández A, Garabaya C, Cal S, López-Otín C. Cloning and enzymatic analysis of 22 novel human ubiquitin-specific proteases. Biochem Biophys Res Commun. 2004;314:54–62. 10.1016/j.bbrc.2003.12.050. 14715245 10.1016/j.bbrc.2003.12.050

[26] Sun T, Liu Z, Yang Q. The role of ubiquitination and deubiquitination in cancer metabolism. Mol Cancer. 2020;19:146. 10.1186/s12943-020-01262-x. 33004065 10.1186/s12943-020-01262-x PMC7529510

[27] Han S, Wang R, Zhang Y, et al. The role of ubiquitination and deubiquitination in tumor invasion and metastasis. Int J Biol Sci. 2022;18:2292–303. 10.7150/ijbs.69411. 35414786 10.7150/ijbs.69411 PMC8990454

[28] Liu J, Cheng Y, Zheng M, et al. Targeting the ubiquitination/deubiquitination process to regulate immune checkpoint pathways. Signal Transduct Target Ther. 2021;6:28. 10.1038/s41392-020-00418-x. 33479196 10.1038/s41392-020-00418-x PMC7819986

[29] Niu Z, Li X, Feng S, et al. The deubiquitinating enzyme USP1 modulates ERα and modulates breast cancer progression. J Cancer. 2020;11:6992–7000. 10.7150/jca.50477. 33123289 10.7150/jca.50477 PMC7591989

[30] Zhang J, Zhang P, Wei Y, et al. Deubiquitylation and stabilization of PTEN by USP13. Nat Cell Biol. 2013;15:1486–94. 10.1038/ncb2874. 24270891 10.1038/ncb2874 PMC3951854

[31] Zhang J, Liu S, Li Q, et al. The deubiquitylase USP2 maintains ErbB2 abundance via counteracting endocytic degradation and represents a therapeutic target in ErbB2-positive breast cancer. Cell Death Differ. 2020;27:2710–25. 10.1038/s41418-020-0538-8. 32327714 10.1038/s41418-020-0538-8 PMC7429833

[32] Cloutier JM, Kunder CA, Charville GW, et al. Nodular fasciitis of the breast: clinicopathologic and molecular characterization with identification of novel USP6 fusion partners. Mod Pathol. 2021;34:1865–75. 10.1038/s41379-021-00844-4. 34099872 10.1038/s41379-021-00844-4

[33] Liu T, Sun B, Zhao X, et al. USP44 + Cancer stem cell subclones contribute to breast Cancer aggressiveness by promoting Vasculogenic Mimicry. Mol Cancer Ther. 2015;14:2121–31. 10.1158/1535-7163.MCT-15-0114-T. 26232424 10.1158/1535-7163.MCT-15-0114-T

[34] Li L, Wang S, Zhou W. Balance Cell apoptosis and pyroptosis of caspase-3-Activating chemotherapy for Better Antitumor Therapy. Cancers (Basel). 2022;15. 10.3390/cancers15010026. 10.3390/cancers15010026 PMC9817729 36612023

[35] Unnisa A, Greig NH, Kamal MA. Inhibition of Caspase 3 and caspase 9 mediated apoptosis: a Multimodal Therapeutic Target in Traumatic Brain Injury. Curr Neuropharmacol. 2023;21:1001–12. 10.2174/1570159X20666220327222921. 35339178 10.2174/1570159X20666220327222921 PMC10227914

[36] Wurstle ML, Laussmann MA, Rehm M. The central role of initiator caspase-9 in apoptosis signal transduction and the regulation of its activation and activity on the apoptosome. Exp Cell Res. 2012;318:1213–20. 10.1016/j.yexcr.2012.02.013. 22406265 10.1016/j.yexcr.2012.02.013

[37] Begum HM, Shen K. Intracellular and microenvironmental regulation of mitochondrial membrane potential in cancer cells. WIREs Mech Dis. 2023;15:e1595. 10.1002/wsbm.1595. 36597256 10.1002/wsbm.1595 PMC10176868

[38] Zhang RX, Liu FF, Lip H, Liu J, Zhang Q, Wu XY. Pharmaceutical nanoformulation strategies to spatiotemporally manipulate oxidative stress for improving cancer therapies - exemplified by polyunsaturated fatty acids and other ROS-modulating agents. Drug Deliv Transl Res. 2022;12:2303–34. 10.1007/s13346-021-01104-3. 35064476 10.1007/s13346-021-01104-3

[39] Gao L, Xu Z, Huang Z, et al. CPI-613 rewires lipid metabolism to enhance pancreatic cancer apoptosis via the AMPK-ACC signaling. J Exp Clin Cancer Res. 2020;39:73. 10.1186/s13046-020-01579-x. 32345326 10.1186/s13046-020-01579-x PMC7187515

[40] Cao Y, Wang J, Tian H, Fu GH. Mitochondrial ROS accumulation inhibiting JAK2/STAT3 pathway is a critical modulator of CYT997-induced autophagy and apoptosis in gastric cancer. J Exp Clin Cancer Res. 2020;39:119. 10.1186/s13046-020-01621-y. 32576206 10.1186/s13046-020-01621-y PMC7310559

[41] Mevissen TET, Komander D. Mechanisms of Deubiquitinase specificity and regulation. Annu Rev Biochem. 2017;86:159–92. 10.1146/annurev-biochem-061516-044916. 28498721 10.1146/annurev-biochem-061516-044916

[42] Wang X, Feng H, Dong W, Wang F, Zhang G, Wu J. Hsa_circ_0008225 inhibits tumorigenesis of glioma via sponging miR-890 and promoting ZMYND11 expression. J Pharmacol Sci. 2020;143:74–82. 10.1016/j.jphs.2020.02.008. 32192854 10.1016/j.jphs.2020.02.008

[43] Cui J, Yuan Y, Shanmugam MK, et al. MicroRNA-196a promotes renal cancer cell migration and invasion by targeting BRAM1 to regulate SMAD and MAPK signaling pathways. Int J Biol Sci. 2021;17:4254–70. 10.7150/ijbs.60805. 34803496 10.7150/ijbs.60805 PMC8579441

[44] Goldhirsch A, Wood WC, Coates AS, Gelber RD, Thürlimann B, Senn HJ. Strategies for subtypes–dealing with the diversity of breast cancer: highlights of the St. Gallen International Expert Consensus on the primary therapy of early breast Cancer 2011. Ann Oncol. 2011;22:1736–47. 10.1093/annonc/mdr304. 21709140 10.1093/annonc/mdr304 PMC3144634

