# Supplementary figures and images for: USP53 Affects the Proliferation and Apoptosis of Breast Cancer Cells by Regulating the Ubiquitination Level of ZMYND11
Source: Biol Proced Online. 2024 Jul 23;26:24. doi: 10.1186/s12575-024-00251-4 (PMC11264418; doi:10.1186/s12575-024-00251-4)

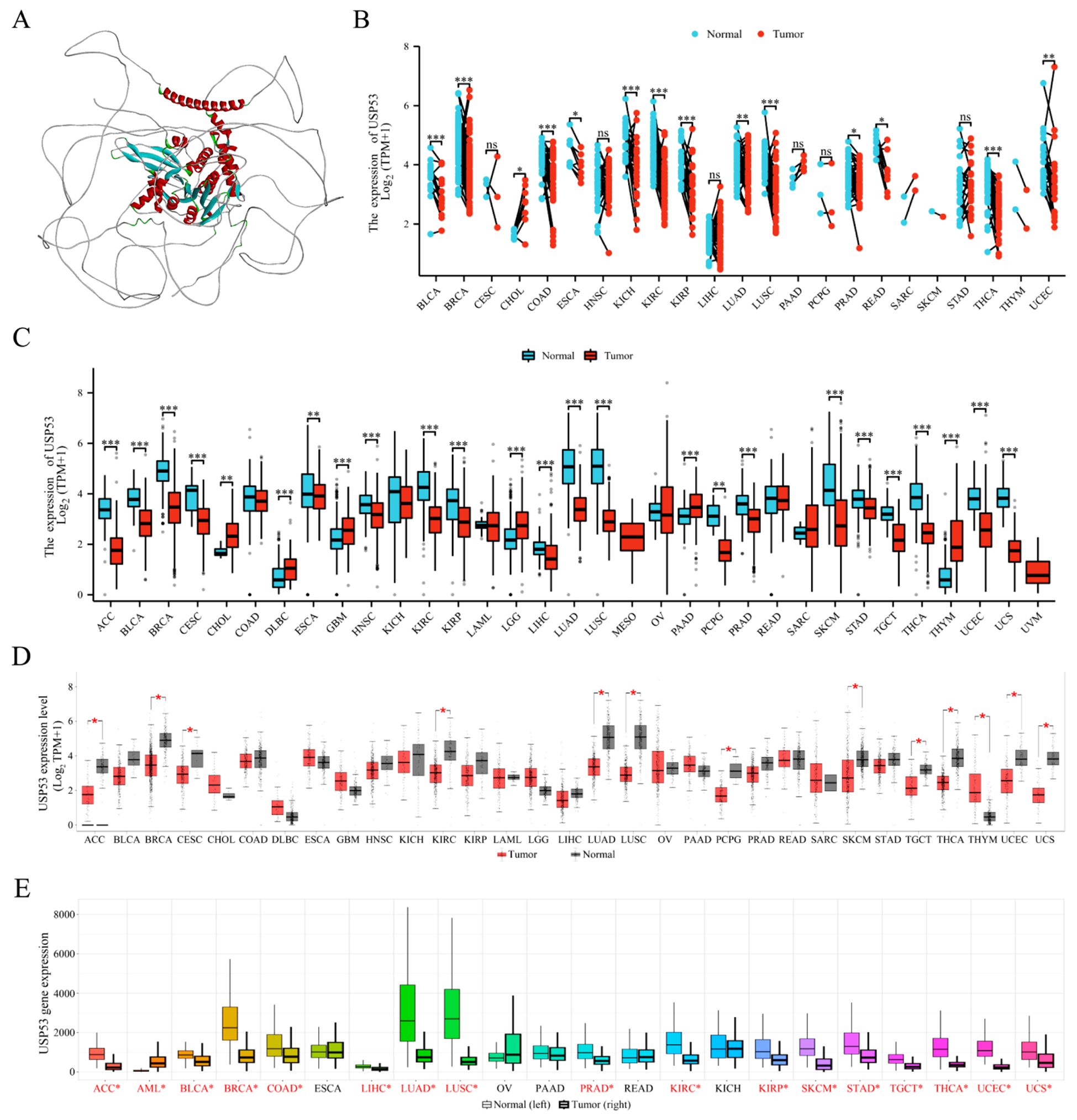

Supplement: Supplementary file 1 — Supplementary Material 1 [file 12575_2024_251_MOESM1_ESM.tif]
