# Supplementary material for: USP53 Affects the Proliferation and Apoptosis of Breast Cancer Cells by Regulating the Ubiquitination Level of ZMYND11
Source: Biol Proced Online. 2024 Jul 23;26:24. doi: 10.1186/s12575-024-00251-4 (PMC11264418; doi:10.1186/s12575-024-00251-4)
Supplement: Supplementary file 2 — Supplementary Material 2 [file 12575_2024_251_MOESM2_ESM.docx]

**Supplemental Material**

Supplementary Table S1. Full names and abbreviations of the 33 types of cancer

| **Abbr** | **Full name** | **Abbr** | **Full name** | **Abbr** | **Full name** |
| --- | --- | --- | --- | --- | --- |
| **ACC** | Adrenocortical cancer | **KIRC** | kidney renal clear cell cancer | **PRAD** | Prostate adenocarcinoma |
| **BLCA** | Bladder urothelial cancer | **KIRP** | Kidney renal papillary cell cancer | **READ** | Rectum adenocarcinoma |
| **BRCA** | Breast invasive carcinoma | **LAML** | Acute myeloid leukemia | **SARC** | Sarcoma |
| **CESE** | Cervical & Endocervical cancer | **LGG** | Brain lower grade glioma | **SKCM** | Skin cutaneous melanom |
| **CHOL** | Cholangiocarcinoma | **LIHC** | Liver hepatocellular cancer | **STAD** | Stomach adenocarcinoma |
| **COAD** | Colon adenocarcinoma | **LUAD** | Lung adenocarcinoma | **TGCT** | Testicular germ cell tumors |
| **DLBC** | Diffuse large B-cell lymphoma | **LUSC** | Lung squamous cell cancer | **THCA** | Thyroid cancer |
| **ESCA** | Esophageal cancer | **MESO** | Mesothelioma | **THYM** | Thymoma |
| **GBM** | Glioblastoma multiforme | **OV** | Ovarian serous cystadenocarcinoma | **UCEC** | Uterine corpus endometrial carcinoma |
| **HNSC** | Head & Neck squamous cell cancer | **PAAD** | Pancreatic adenocarcinoma | **USC** | Uveal melanoma |
| **KICH** | Kidney chromophobe | **PCPG** | Pheochromocytoma and Paraganglioma | **UVM** | Uveal melanoma |
